# The Regulation of Frontal Cortex Cholesterol Metabolism Abnormalities by NR3C1/NRIP1/NR1H2 Is Involved in the Occurrence of Stress-Induced Depression

**DOI:** 10.3390/ijms25158075

**Published:** 2024-07-24

**Authors:** Rui Shi, Yingmin Li, Weihao Zhu, Hongjian Xin, Huihuang Yang, Xiaowei Feng, Zhen Wang, Shujin Li, Bin Cong, Weibo Shi

**Affiliations:** Collaborative Innovation Center of Forensic Medical Molecular Identification, Hebei Key Laboratory of Forensic Medicine, Department of Forensic Medicine, Hebei Medical University, Shijiazhuang 050017, China; 22033100275@stu.hebmu.edu.cn (R.S.); 16000557@hebmu.edu.cn (Y.L.); 22031100049@stu.hebmu.edu.cn (W.Z.); 22033100279@stu.hebmu.edu.cn (H.X.); 22033100274@stu.hebmu.edu.cn (H.Y.); 22033100268@stu.hebmu.edu.cn (X.F.); 22033100273@stu.hebmu.edu.cn (Z.W.); shujinli@hebmu.edu.cn (S.L.)

**Keywords:** depression, cholesterol metabolism, frontal cortex, NR3C1/NRIP1/NR1H2 pathway

## Abstract

Stress-induced alterations in central neuron metabolism and function are crucial contributors to depression onset. However, the metabolic dysfunctions of the neurons associated with depression and specific molecular mechanisms remain unclear. This study initially analyzed the relationship between cholesterol and depression using the NHANES database. We then induced depressive-like behaviors in mice via restraint stress. Applying bioinformatics, pathology, and molecular biology, we observed the pathological characteristics of brain cholesterol homeostasis and investigated the regulatory mechanisms of brain cholesterol metabolism disorders. Through the NHANES database, we initially confirmed a significant correlation between cholesterol metabolism abnormalities and depression. Furthermore, based on successful stress mouse model establishment, we discovered the number of cholesterol-related DEGs significantly increased in the brain due to stress, and exhibited regional heterogeneity. Further investigation of the frontal cortex, a brain region closely related to depression, revealed stress caused significant disruption to key genes related to cholesterol metabolism, including HMGCR, CYP46A1, ACAT1, APOE, ABCA1, and LDLR, leading to an increase in total cholesterol content and a significant decrease in synaptic proteins PSD-95 and SYN. This indicates cholesterol metabolism affects neuronal synaptic plasticity and is associated with stress-induced depressive-like behavior in mice. Adeno-associated virus interference with NR3C1 in the prefrontal cortex of mice subjected to short-term stress resulted in reduced protein levels of NRIP1, NR1H2, ABCA1, and total cholesterol content. At the same time, it increased synaptic proteins PSD95 and SYN, effectively alleviating depressive-like behavior. Therefore, these results suggest that short-term stress may induce cholesterol metabolism disorders by activating the NR3C1/NRIP1/NR1H2 signaling pathway. This impairs neuronal synaptic plasticity and consequently participates in depressive-like behavior in mice. These findings suggest that abnormal cholesterol metabolism in the brain induced by stress is a significant contributor to depression onset.

## 1. Introduction

In the fourth century BC, Hippocrates proposed that “Sadness and fear, if prolonged, can lead to melancholy.” This illustrates that depression has been one of the most prevalent mental disorders throughout history. According to World Health Organization statistics, approximately 3.8% of the global population suffers from depression, and it is projected to become the leading cause of global disease burden by 2030. In China, 95 million people have depression, with a lifetime prevalence rate of major depressive disorder at 6.8%, and about 280,000 people commit suicide annually, with approximately 40% suffering from depression [1,2]. However, the etiology and pathogenesis of this disease remain unclear. Genetic factors [3], neurological factors [4,5,6], and social psychological factors [7,8] play significant roles in depression occurrence, and stress-induced changes in central nervous system function and metabolism are major causes [9]. Studies show individuals experiencing adverse events (e.g., unemployment, death, abuse, or other trauma) early in life are at higher risk for depression [10,11,12]. Moreover, people with depression often experience more stressful events before their depressive episode compared to healthy individuals [13,14,15,16]. Therefore, stress is widely recognized as a potential risk factor in depression development and progression.

Previous studies have demonstrated that stress can lead to dysregulation of lipid metabolism in various organs, including skeletal muscle, bone, liver, and pancreas [17]. However, the brain, as the organ with the richest lipid concentration, has rarely been reported in this context. Cholesterol, as an important type of lipid, is indispensable to cellular tissues. It interacts with neighboring lipid molecules to regulate membrane rigidity, fluidity, and permeability, and binds with transmembrane proteins to maintain or alter their conformation, influencing cellular signaling pathways. Cholesterol also participates in the exchange of substances between cells and the external environment and serves as a precursor for bile acids, vitamin D, and steroid hormone synthesis [18,19]. Cholesterol is most abundant in the brain and neural tissues. Since it cannot freely cross the blood–brain barrier, nearly all cholesterol in the brain is synthesized by astrocytes in situ and used to maintain neural protrusions and synaptic connections [20]. 

Brain cholesterol biosynthesis is a multi-step process. First, acetyl-Coenzyme A is converted into 3-hydroxy-3-methylglutaryl-Coenzyme A (HMG-CoA), then through HMG-CoA reductase, it is converted into mevalonic acid, followed by multiple reactions, it is transformed into lanosterol. Cholesterol in the brain is generated from various sterol intermediates, depending on the cell types. The synthesized cholesterol is bound to APOE and secreted extracellularly through ABC transporters on the cell membrane. It can be absorbed by two types of LDL receptors (LDLR and LRPs) and transported to neurons, where it is utilized for membrane turnover, repair, myelin formation, synaptogenesis, and neurotransmitter release. Excess cholesterol can be esterified by cholesterol acyltransferase 1 (ACAT1/SOAT1) and stored in lipid droplets. It can also be converted into 24-hydroxycholesterol (24-OHC) by 24-hydroxylase (CYP46A1). Unlike cholesterol, 24-OHC diffuses across the blood–brain barrier under the influence of concentration gradients and enters the systemic circulation [21]. Research has shown that in stressed rats, serum cholesterol levels increase, hippocampal synaptic plasticity is impaired, and the rats develop cognitive dysfunction, suggesting that disrupted cholesterol metabolism may contribute to CUMS-induced cognitive impairments [22]. Stress also enhances the transcriptional activity of cholesterol synthesis genes LXR, SREBP1c, and ChREBP, disrupting brain lipid metabolism and affecting brain function [23]. However, the specific regulatory mechanisms of cholesterol metabolism in the brain after stress, as well as the molecular networks linking cholesterol metabolism disruption and stress-induced damage, remain unclear and are seldom reported.

Against the backdrop of depression, this study focuses on brain cholesterol metabolism. It utilizes NHANES data for prospective analysis of the relationship between cholesterol-related indicators and depression. Employing bioinformatics techniques, it identifies differential small molecule markers in stress-induced injuries. From a new perspective, the study explores the molecular interactions between stress-induced damage and cholesterol metabolism, elucidating the roles of NR3C1/NRIP1/NR1H2 in the mechanisms underlying depression-like behavior induced by short-term restraint stress. 

## 2. Results

### 2.1. Abnormal Cholesterol Levels in the Serum of Depression Patients

Data were extracted from the National Health and Nutrition Examination Survey (NHANES) database for 48,715 subjects aged 20–85 years. This included basic information, laboratory cholesterol-related indicators (total cholesterol, high-density lipoprotein (HDL), etc.), and depression scale scores. The aim was to assess if cholesterol is a risk factor for depression. After excluding 22,667 subjects with missing or incorrect data, 26,048 subjects were analyzed. Depression was defined as a PHQ-9 score ≥ 10. A single-factor logistic regression model was used to determine relationships between gender, age, race, education level, body mass index, income-to-poverty ratio, and total cholesterol with depression risk. The results showed age and race were not significantly associated with depression. Gender, education level, body mass index, income-to-poverty ratio, HDL, and total cholesterol were all risk factors for depression (*p* < 0.05) (Table 1), suggesting abnormal cholesterol-related indicators may play a role in depression development.

### 2.2. Dysregulation of Cholesterol Metabolism in the Mouse Brain under Stress

**Figure 1:**
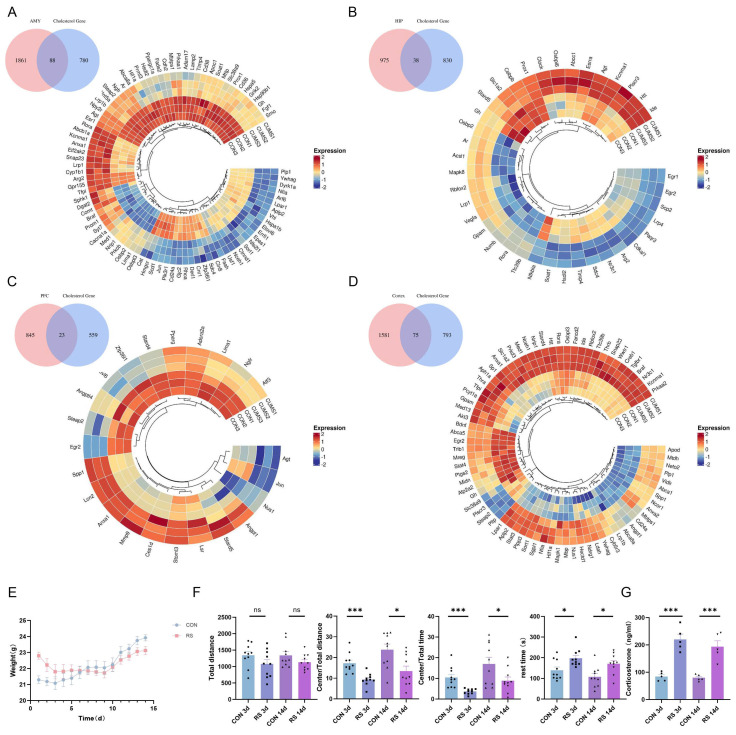
Dysregulation of Cholesterol Metabolism in the Mouse Brain under Stress. (**A**–**D**) Analysis of stress−induced changes in cholesterol-related genes across different brain regions. Heatmap of DEGs with increasing and decreasing trend. Each column represents a sample, and each gene is visualized in a row. Red indicates a high abundance of the gene, and blue indicates a relatively low abundance of the gene. (**E**) Changes in body weight of mice. (**F**) In the SPT, the model group mice showed a significant decrease in the distance traveled in the central zone, and time spent in the central zone. In the TST, the immobility time of the modeling mice significantly increased (from 13 mice, *n* = 13). (**G**) Plasma cortisol measured in mice. Values are expressed as the mean ± SEM, * *p* < 0.05, *** *p* < 0.001 vs. the control group.

A systematic analysis of stress-induced changes in cholesterol-related genes across different brain regions was performed using the GSE151807 dataset, which included the amygdala, hippocampus, prefrontal cortex, and cortex of stressed mice. A total of 1949 differentially expressed genes (DEGs) were identified in the amygdala, 1013 in the hippocampus, 582 in the prefrontal cortex, and 1656 in the cortex. Intersecting these DEGs with 868 cholesterol-related genes revealed 88 cholesterol-related DEGs in the amygdala (24 up-regulated and 64 down-regulated), 38 in the hippocampus (20 up-regulated and 18 down-regulated), 23 in the prefrontal cortex (13 up-regulated and 10 down-regulated), and 75 in the cortex (61 up-regulated and 14 down-regulated). The heatmap of DEG expression levels across different brain regions showed significant differences between the control and stressed groups, as well as substantial differences among different brain regions (Figure 1A–D). This suggests that stress can lead to dysregulation of cholesterol metabolism in the brain, with regional heterogeneity in the extent of dysregulation.

**Figure 2:**
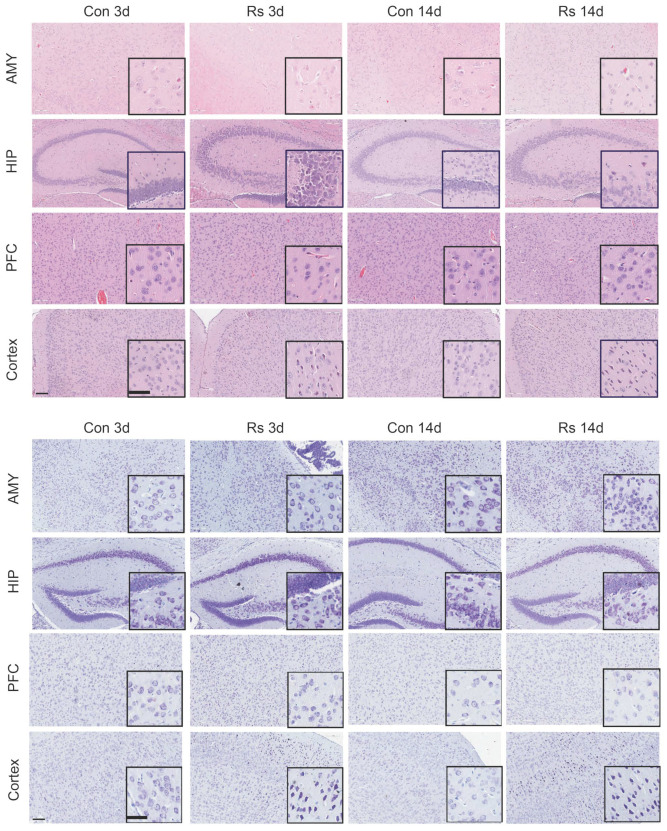
Dysregulation of Cholesterol Metabolism in the Mouse Brain under Stress. HE staining and thionine staining revealed edema in the amygdala, hippocampus, prefrontal cortex, and cortex of stressed mice.The enlarged image is located in the bottom right corner boxes. The scale bars in the figure are all 100 µm.

**Figure 3:**
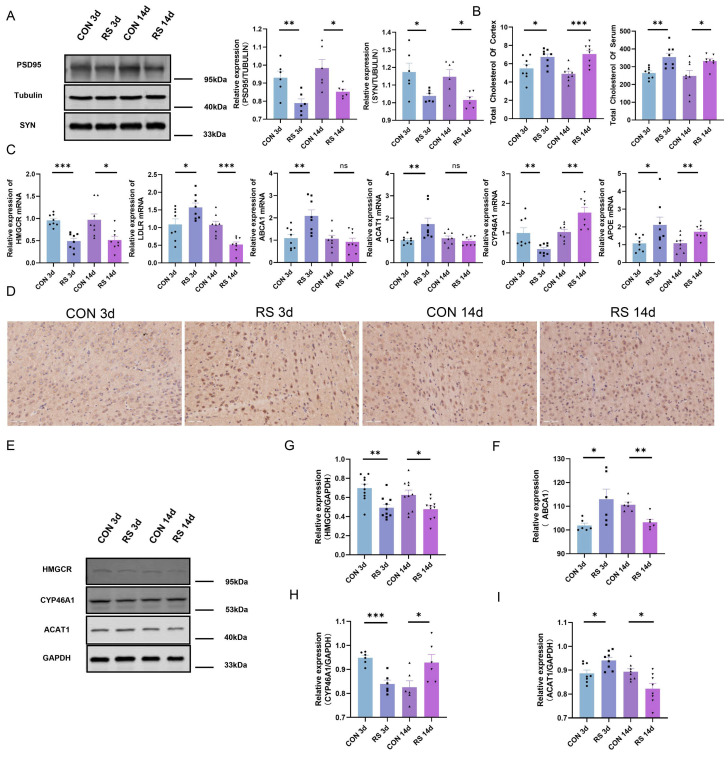
Dysregulation of Cholesterol Metabolism in the Mouse Brain under Stress. (**A**) Representative Western blot images and densitometric quantification of PSD-95 and SYN in the frontal cortex (*n* = 6). (**B**) Total cholesterol content in serum and frontal cortex of mice (from 4 mice, repeated twice, *n* = 8). (**C**) The mRNA level of HMGCR, LDLR, ABCA1, ACAT1, CYP46A1, APOE (from 4 mice, repeated twice, *n* = 8). (**D**,**F**) Representative micrographs and quantitative analysis of ABCA1 immunohistochemical staining in the frontal cortex (from 3 mice, repeated twice, *n* = 6). (**E**,**G**–**I**) Representative Western blot images and densitometric quantification of HMGCR, CYP46A1, and ACAT1 in the frontal cortex (from 6 mice, repeated tests were conducted with the smallest statistically significant value of *n*, including HMGCR (*n* = 10), CYP46A1 (*n* = 6), and ACAT1 (*n* = 8)). Values are expressed as the mean ± SEM, * *p* < 0.05, ** *p* < 0.01, *** *p* < 0.001 vs. the control group.

To further explore the relationship between dysregulation of the cholesterol metabolism in the brains of stressed mice and depressive-like behavior, we attempted to establish long-term and short-term restraint stress mouse models. Fourteen-day weight data showed the control mice’s weight gradually increased, while the stressed mice’s weight decreased during the first nine days and then gradually increased, but remained lower than the control mice (*p* < 0.05) (Figure 1E). Behavioral tests revealed no significant differences in total activity distance among groups, but the ratio of central activity distance to total distance, as well as the ratio of central activity time to total time, was significantly reduced in both long-term and short-term stressed mice (*p* < 0.05). Additionally, the immobility time during tail suspension was significantly increased, indicating both long-term and short-term stressed mice exhibited depressive-like and anxious-like behaviors (Figure 1F). Furthermore, serum cortisol levels in long-term and short-term stressed mice were significantly higher than in the control group (Figure 1G). These results indicate long-term and short-term restraint stress models were successfully established, and mice exhibited depressive-like behavior. HE staining and thionine staining revealed edema in the amygdala, hippocampus, prefrontal cortex, and cortex of stressed mice, as well as neuronal pyknosis and obvious eosinophilic changes, with the most significant pathological changes observed in the cortex (Figure 2). Consequently, the frontal cortex, closely related to depressive-like behavior in mice, was selected for further research. Western blotting analysis showed expression of PSD-95 and SYN proteins in the frontal cortex of long-term and short-term stressed mice was reduced, indicating stress can damage the synaptic plasticity of neurons in the frontal cortex of mice (Figure 3A). Additionally, the detection of total cholesterol content in the serum and frontal cortex of mice revealed cholesterol levels increased after long-term and short-term stress (Figure 3B). Consequently, we speculate dysregulation of cholesterol metabolism in the frontal cortex of stressed mice may damage the synaptic plasticity of neurons, thereby leading to the production of depressive-like behavior.

It has been reported in previous studies that the levels of srebp1, hmgcr, and cyp51 genes are all significantly increased in stress-responsive animals after FSS [24]. To elucidate changes in cholesterol metabolism in the frontal cortex after stress, we conducted mRNA and protein level analyses of key metabolic proteins. In the frontal cortex of stressed mice after 3 days, relative mRNA contents of HMGCR and CYP46A1 decreased, while ACAT1 increased. These trends were consistent with protein level changes. In contrast, the relative mRNA contents of APOE, ABCA1, and LDLR increased, and the immunohistochemical results for ABCA1 were in agreement. This suggests that in the frontal cortex of short-term stressed mice, cholesterol biosynthesis decreased, cholesterol transport increased, cholesterol was converted to cholesteryl esters within neurons, and the storage of these esters in lipid droplets was enhanced. For mice stressed for 14 days, the relative mRNA contents of HMGCR and LDLR decreased, while CYP46A1 and APOE increased, with no changes in ACAT1 or ABCA1. Protein level detection showed HMGCR and CYP46A1 had consistent trends with mRNA changes, but ACAT1 and ABCA1 protein expression decreased, indicating that in the frontal cortex of long-term stressed mice, cholesterol biosynthesis decreased, transport to neurons decreased, and cholesterol was mainly converted to 24-OHC within neurons (Figure 3C–I). Studies have shown that under normal conditions or during the early stages of neurodegenerative disease, it would seem that low levels of 24S-OHC act protectively, but that as 24S-OHC levels increase, a point is reached at which 24S-OHC no longer acts protectively but instead functions causally to induce cell death [25]. Collectively, these findings suggest long-term and short-term stress significantly impact cholesterol metabolism direction in the frontal cortex.

### 2.3. NR3C1/NRIP1/NR1H2 Pathway Involved in Depression-like Behavior Induced by Short-Term Stress

**Figure 4:**
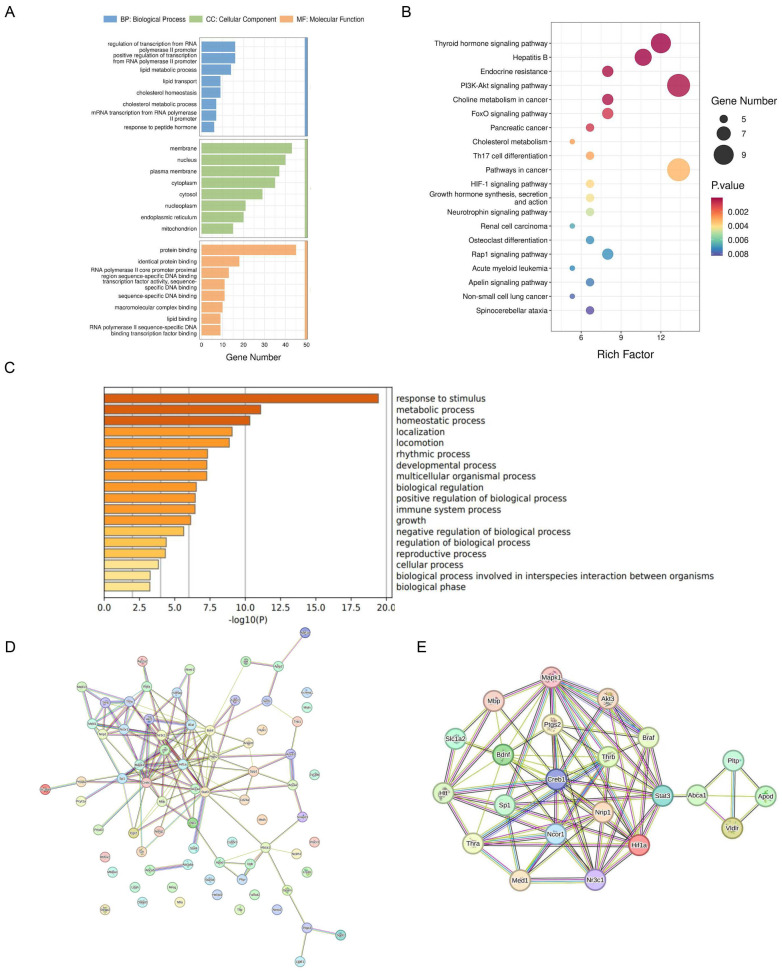
NR3C1/NRIP1/NR1H2 Pathway Involved in Depression-Like Behavior Induced by Short-Term Stress. (**A**) GO pathway enrichment results from intersecting genes. (**B**) Signal pathway of KEGG enrichment analysis. (**C**) The enriched terms in the Metascape database. (**D**) PPI network of 75 cholesterol−related genes in the STRING database. The color of the lines indicates the type of interaction evidence. (**E**) PPI network of 22 hub target genes.

**Figure 5:**
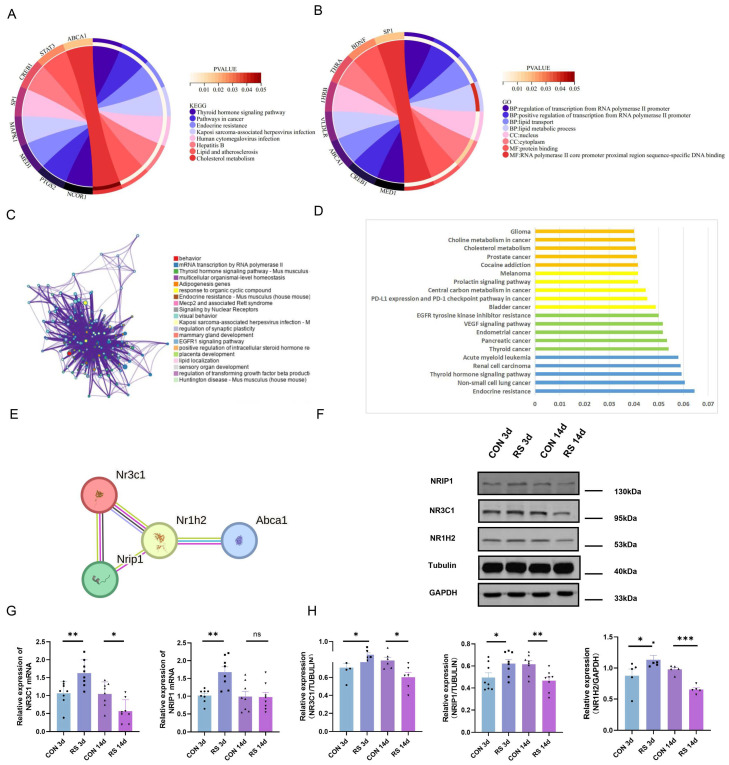
NR3C1/NRIP1/NR1H2 Pathway Involved in Depression−Like Behavior Induced by Short−Term Stress. (**A**,**B**) GO and KEGG enrichment analysis in the KOBAS database. (**C**) Enriched terms for central node genes in the Metascape database. (**D**) Enriched terms for central node genes in the KOBAS database. (**E**) PPI network of NR3C1, NRIP1, NR1H2 and ABCA1. (**G**) The mRNA level of NR3C1 and NRIP1 (from 4 mice, repeated twice, *n* = 8). (**F**–**H**) Representative Western blot images and densitometric quantification of NR3C1, NRIP1, and NR1H2 in the frontal cortex (from 6 mice, repeated tests were conducted with the smallest statistically significant value of *n*, including NR3C1 (*n* = 6), NRIP1 (*n* = 8), and NR1H2 (*n* = 5)). Values are expressed as the mean ± SEM, * *p* < 0.05, ** *p* < 0.01, *** *p* < 0.001 vs. the control group.

To elucidate the specific molecular interaction network of stress-cholesterol metabolism disorder-depressive-like behavior, the cholesterol-related DEGs from the cortex were uploaded to the Database for Annotation, Visualization and Integrated Discovery (DAVID) for functional enrichment analysis. Significant enrichment was found in biological processes such as cholesterol homeostasis, lipid metabolic process, and lipid binding (Figure 4A,B). Similarly, the Metascape database also enriched cholesterol metabolism-related biological functions (Figure 4C), suggesting that under stress conditions, the cholesterol metabolism pathway may play an important role. To identify core molecular markers, the cholesterol-related DEGs were analyzed using STRING to construct a protein–protein interaction (PPI) network. A total of 75 DEGs appeared in the PPI network, with 141 protein interaction lines (Figure 4D). The PPI enrichment *p*-value was <1.0 × 10^−16^. Using the MCODE application function of Cytoscape 3.9.1 software, three gene clusters with high intersection scores were identified, with scores of 7.750, 4.250, and 4.000, respectively. These clusters contained 31, 17, and 6 protein interaction lines, with 9, 9, and 4 central nodes, respectively. The 22 central node genes were defined as core DEGs (19 up-regulated and 3 down-regulated) (Figure 4E). Gene ontology (GO) functional analysis and Kyoto Encyclopedia of Genes and Genomes (KEGG) pathway enrichment results showed that they mainly participate in biological processes such as cholesterol metabolism, lipid transport, and lipid metabolic process (Figure 5A,B). Metascape and KOBAS databases also enriched cholesterol metabolism-related pathways (Figure 5C,D).

Further analysis of the core DEGs revealed that NR3C1, encoding one of the glucocorticoid receptors, has been demonstrated in previous studies to be significantly associated with depression [26]. Nuclear receptor-interacting protein 1 (NRIP1, also known as RIP140) is a nuclear protein that specifically interacts with the hormone-dependent activation domain AF2 of nuclear receptors, regulating the transcriptional activation of estrogen receptors and steroid receptors (such as NR3C1, NR3C2, and ESR1) [27]. Simultaneously, NRIP1 can interact with many ligand-binding nuclear receptors, such as peroxisome proliferator-activated receptors (PPARs), liver X receptors (LXRs), and estrogen-related receptors (ERRs), and is particularly important in regulating lipid and glucose metabolism. Among these, LXR, as a nuclear receptor expressed in various cells in the human body, can affect the body’s metabolism by regulating downstream cholesterol metabolism-related genes (such as ABCA1 and SREBP-1C) [28]. The results of the differential analysis also showed that NR3C1 and NRIP1 exhibited higher fold changes before and after stress, and the PPI network revealed a strong interaction between NR3C1, NRIP1, and NR1H2 (Figure 5E). Therefore, it is speculated that these three may play important roles in the induction of depressive-like behavior by cholesterol metabolism disorders. To this end, the expression of NR3C1, NRIP1, and NR1H2 in the frontal cortex of long-term and short-term stressed mice was detected. The results showed that in the frontal cortex of stressed mice after 3 days, the mRNA and protein levels of NR3C1 and NRIP1 increased, and the protein expression of NR1H2 also increased. In the frontal cortex of stressed mice after 14 days, the expression levels of NR3C1, NRIP1, and NR1H2 all decreased (Figure 5F–H). This suggests that the role of this pathway in the process of cholesterol metabolism disorders caused by long-term and short-term stress may be different.

**Figure 6:**
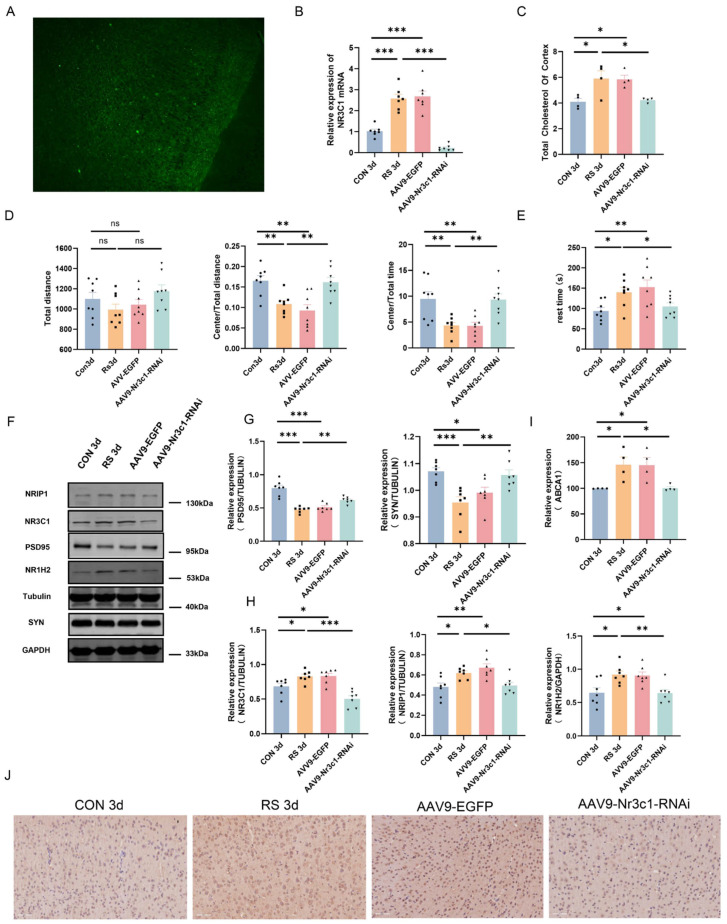
NR3C1/NRIP1/NR1H2 Pathway Involved in Depression−Like Behavior Induced by Short-Term Stress. (**A**) Immunofluorescence staining was performed in the frontal cortex after injecting inhibitory virus AAV9−Nr3c1−RNAi. (**B**) The mRNA level of NR3C1 (from 4 mice, repeated tests were conducted with the smallest statistically significant value of *n*, *n* = 7). (**C**) Total cholesterol content in the frontal cortex of mice (from 4 mice, *n* = 4). (**D**,**E**) In the OFT, the ratio of central activity distance to total distance, as well as the ratio of central activity time to total time in the SPT increased. The immobility time in the TST decreased (from 8 mice, *n* = 8). (**F**–**H**) Representative Western blot images and densitometric quantification of NR3C1, NRIP1, NR1H2, PSD-95, and SYN in the frontal cortex (from 4 mice, repeated tests were conducted with the smallest statistically significant value of *n*, *n* = 7). (**I**,**J**) Representative micrographs and quantitative analysis of ABCA1 immunohistochemical staining in the frontal cortex (from 4 mice, *n* = 4). Values are expressed as the mean ± SEM, * *p* < 0.05, ** *p* < 0.01, *** *p* < 0.001 vs. the control group.

Subsequently, the mechanism of the NR3C1/NRIP1/NR1H2 pathway in depressive-like behavior induced by short-term stress was further investigated. By injecting inhibitory virus AAV9-Nr3c1-RNAi into the bilateral cortices of mice to interfere with NR3C1 expression, its effect on the expression of NRIP1, NR1H2, and depressive-like behavior was explored. After virus injection and recovery for 4 weeks, a short-term restraint stress model was established. Immunofluorescence observation and NR3C1 mRNA detection showed that the adenovirus model was successfully constructed (Figure 6A,B). After specifically knocking down NR3C1, the total cholesterol content decreased, indicating that cholesterol metabolism dysregulation was alleviated (Figure 6C). The protein expression levels of NRIP1, NR1H2, and ABCA1 all decreased (Figure 6F,H–J), suggesting that NRIP1/NR1H2 may be regulated by NR3C1. Additionally, the expression levels of PSD-95 and SYN proteins increased (Figure 6F,G). The ratio of central activity distance to total distance, as well as the ratio of central activity time to total time, increased in the OFT, and the immobility time decreased in the TST. The data indicate that the depressive-like behavior of stressed mice was relieved (Figure 6D,E). Based on these results, it is speculated that short-term stress can activate the NR3C1/NRIP1/NR1H2 signaling pathway, leading to cholesterol metabolism dysregulation, synaptic plasticity damage, and subsequently, the manifestation of depressive-like behavior.

## 3. Discussion

Depression is one of the most common and harmful mental illnesses, causing significant social function impairment in most patients, including emotional lowness, insomnia or hypersomnia, appetite changes, fatigue, feelings of worthlessness or lack of self-worth, difficulty concentrating, and, in severe cases, hallucinations, delusions, and even suicidal behavior [29,30]. Stress is a high-risk factor for depression, leading to significant dysregulation of the hypothalamic-pituitary-adrenal axis [31]. This dysregulation is primarily manifested by increased corticotropin-releasing hormone in the hypothalamic paraventricular nucleus, which in turn leads to increased downstream glucocorticoids. These hormones significantly affect metabolic functions. For example, elevated glucocorticoids cause neuroendocrine and neurotransmitter changes, leading to adaptive changes at the molecular, cellular, and synaptic levels. These changes are involved in depression development [32,33]. However, no reports show stress-induced glucocorticoid elevation leads to brain cholesterol metabolism dysregulation. The relationship between cholesterol metabolism disorders and depression after stress and specific molecular mechanisms remains unclear.

Addressing a knowledge gap, this study systematically analyzed data from 26,048 subjects using the NHANES database and found no significant association between LDL and depression. However, HDL, total cholesterol, and triglycerides were identified as independent risk factors for depression. This finding is consistent with the results reported by Wagner, C.J et al. [34]. Additionally, Qi, X. et al. also found that compared to the lowest reference group for non-high-density lipoprotein cholesterol to high-density lipoprotein cholesterol ratio (NHHR), after full adjustment, participants in the fourth quartile had a significantly increased risk of depression, indicating NHHR was significantly associated with a higher risk of depression [35]. These results all suggest abnormalities in cholesterol-related indicators may play a role in depression’s occurrence and development. However, central cholesterol’s impact on depression remains relatively unknown.

Cholesterol is a component of neuronal cell membranes and may play an important role in neurotransmission and neuroprotection. Therefore, changes in cholesterol levels may relate to normal neurotransmitter function and neuronal health, affecting mental health. Evidence suggests several neurodegenerative diseases, such as Alzheimer’s disease and Huntington’s disease, are associated with disrupted cholesterol homeostasis [36,37,38]. Furthermore, studies show that dysregulated cholesterol metabolism can damage synaptic plasticity, leading to learning and memory impairments [39]. Therefore, we further analyzed changes in cholesterol-related genes across different brain regions after stress. By intersecting the DEGs of stressed mice with cholesterol-related genes, we identified cholesterol-related DEGs. DEGs showed stress can lead to dysregulation of cholesterol metabolism in the brain, with regional heterogeneity. HE and thionine staining revealed cortex tissue was most significantly affected by edema, neuronal pyknosis, and eosinophilic changes. Moreover, there is a large body of research confirming the frontal cortex is a sensitive brain region for depression [40,41]. Therefore, the frontal cortex was chosen as the main research subject.

HMGCR is a key enzyme for cholesterol synthesis in astrocytes [42,43], and astrocytes secrete cholesterol mainly through the apolipoprotein APOE [44]. It binds to ABCA transporters on the membrane, such as ABCA1, and is secreted into the extracellular space. LDLR mediates the uptake of cholesterol by neurons, thereby maintaining the formation of neurites and synaptic connections [45]. CYP46A1 [46] and ACAT1 are the key enzymes for cholesterol to be metabolized into 24-OHC and cholesteryl esters, respectively [47]. These are all key proteins in the cholesterol metabolism pathway. Therefore, we detected the expression of these key proteins in the frontal cortex of mice and found that in the cortex of stressed mice after 3 days, cholesterol biosynthesis decreased, cholesterol transport increased, and excessive cholesterol in neurons was converted into cholesteryl esters, which were then stored in lipid droplets. In the cortex of stressed mice after 14 days, cholesterol biosynthesis decreased, and the amount of cholesterol transported to neurons decreased. Cholesterol in neurons was mainly converted into 24-OHC. Additionally, the expression of synaptic plasticity proteins PSD-95 and SYN in the frontal cortex of stressed mice decreased. We believe that the depressive-like behavior induced by long-term and short-term restraint stress in mice may be achieved by disrupting cholesterol metabolism homeostasis and damaging synaptic plasticity in neurons.

To elucidate the specific molecular mechanisms of cholesterol metabolism in depressive-like behavior in mice, based on previous bioinformatics analysis, we selected NR3C1, NRIP1, and ABCA1 from 22 core DEGs for study [28,48]. After detecting expression levels, all three significantly increased in short-term stress, but exhibited opposite expression trends in long-term stress. We speculate the NR3C1/NRIP1/NR1H2 pathway plays distinct roles in short- and long-term stress. By interfering with NR3C1 using an adenovirus, we found expression of NRIP1, NR1H2, and ABCA1, as well as total cholesterol levels, decreased, while expression of PSD-95 and SYN increased. Consequently, depressive-like behavior in mice was alleviated. The results above imply that short-term stress may cause dysregulation of cholesterol metabolism by activating the NR3C1/NRIP1/NR1H2 signaling pathway, leading to impairment of neuronal synaptic plasticity, and thus participating in depressive-like behavior in mice.

## 4. Materials and Methods

### 4.1. Experimental Animals and Model Establishment

Healthy male C57BL/6 mice, aged 7–8 weeks and weighing 22 ± 2 g, were purchased from Beijing Vital River Laboratory Animal Technology Co., Ltd (Beijing, China). They were housed at 23 ± 2 °C, 50% humidity under a 12 h light-dark cycle with free access to food and water. After 7 days of acclimatization, 52 mice were randomly divided into the following groups: restraint stress for 3 days, restraint stress for 14 days, and their respective normal control groups, with 13 mice per group. Mice in the stress groups were restrained in a narrow space within a restraint tube for 6–8 h daily at random times, and model establishment was performed as previously described [49]. Mice in the control groups were free to move except for being deprived of food and water during the same period. Following model completion, mice were anesthetized with an intraperitoneal injection of 2% pentobarbital sodium (3.2 mL/kg), and blood was collected from the orbital sinus. They were then euthanized quickly, and brain tissues were extracted. Paraffin sections were made from the brain tissues of 5 mice from each group, while the remaining tissues were rapidly frozen in liquid nitrogen and stored at −80 °C for future analysis.

Thirty-two healthy male C57BL/6 mice were randomly divided into a normal group, a stress group (restrained stress for three days), a virus group (in which inhibitory virus AAV9-Nr3c1-RNAi was injected into the bilateral cortical areas of the mice, with coordinates at AP: −2.18 mm; ML: −1.82 mm; DV: 0.88 mm), and an empty virus vector group (CON305 (type 9) (U6-MCS-CAG-EGFP) was used as a negative control virus injected into the bilateral cortical areas of the mice). There were eight mice in each group.

### 4.2. Open Field Test and Tail Suspension Test

The open field test was conducted in a well-sound-insulated room, using a square arena measuring 40 cm × 40 cm × 40 cm. Each mouse’s movement trajectory was continuously recorded with a video camera throughout the 5 min duration. The central area was defined as the four inner squares, with the rest of the arena considered peripheral. Mice were allowed a 2 h acclimation period before testing. The arena was cleaned with 75% alcohol to eliminate olfactory cues. The time spent in the central area, total distance moved, and total movement time were recorded for each mouse.

For the tail suspension test, the tail of each experimental mouse was fixed with medical tape, approximately 1 cm from the tip of the tail, so that the head was hanging downward. The mice were then videotaped for 5 min, and the duration of immobility and struggling behavior was recorded.

### 4.3. HE Staining and Thionine Staining

The mouse frontal cortex tissue was fixed in 4% paraformaldehyde solution for more than 24 h, embedded in paraffin, and sectioned at about 4 μm thickness. For HE staining, the sections were dehydrated, dewaxed, stained with hematoxylin for a few seconds, washed with tap water, differentiated with hydrochloric acid-alcohol, counterstained with eosin for a few seconds, dehydrated, cleared, and mounted with neutral resin under a microscope.

For thionine staining, paraffin sections were dehydrated and dewaxed, then placed in a preheated (60 °C) thionine staining solution for 1 h. The sections were rinsed with deionized water to remove excess dye, differentiated with 95% alcohol for a few seconds (under microscopic observation), dehydrated, cleared, and mounted with a neutral resin.

### 4.4. Enzyme-Linked Immunosorbent Assay

Blood samples were centrifuged at 4 °C at 1000–2000× *g* for 10 min to separate serum (acidified). Standard solution concentration was established by diluting the standard (STANDARD) per label volume with sample diluent and mixing thoroughly. Dilutions were 1:2, 1:4, 1:8, 1:16, and 1:32 of starting concentration. Samples or standard concentrations were added to respective wells at 100 μL/well, followed by 50 μL/well horseradish peroxidase (HRP)-labeled corticosterone conjugate and mixing. Reaction wells were covered with a sealing membrane (white) and incubated at room temperature away from light for 120 min. After five washes, 100 μL TMB substrate solution was added per well, covered with a sealing membrane (white), and incubated at room temperature away from light for 15–20 min. The reaction was stopped by adding 50 μL stopping solution per well, mixing immediately, and measuring absorbance at 450 nm (A450).

### 4.5. Bioinformatics Data Analysis

From the NCBI database, 868 cholesterol-related genes were collected. Gene expression data for different brain regions (amygdala, hippocampus, prefrontal cortex, and cortex) of mice after stress was obtained from the GSE151807 gene expression profile. For each brain region, 3 brain samples from control mice and 3 brain samples from stressed mice were selected, totaling 6 samples. The GEO2R analysis tool was used to identify DEGs, with the screening criteria of |logFC| ≥ 0.5 and *p*-value < 0.05. The Lianchuan Cloud Platform was used to filter the DEGs that intersected between GSE151807 and the cholesterol-related genes, and a heatmap was generated. The DAVID database (https://david.ncifcrf.gov; (9 November 2023)) was used to perform GO functional analysis and KEGG pathway enrichment analysis on the common intersecting DEGs. The relevant analysis method was performed as previously described [50]. Additionally, Metascape and KOBAS were used for pathway enrichment analysis of the DEGs, with a screening criterion of *p* < 0.05. The STRING tool (https://string-db.org/; (11 November 2023)) was used to construct a PPI network diagram, and Cytoscape 3.9.1 was then used to filter out the core DEGs.

### 4.6. Immunohistochemical Staining

The tissue sections undergo routine deparaffinization, hydration, and immersion in distilled water. High-temperature high-pressure repair (using citrate or EDTA solution) is performed, followed by natural cooling and washing with PBS for 5 min three times. The sections are then placed in 3% H_2_O_2_ at room temperature for 30 min, washed with PBS for 5 min three times, and incubated with goat serum at 37 °C for 40 min for blocking. Primary antibody against ABCA1 (diluted 1:200) is added, and the sections are incubated overnight at 4 °C, followed by washing with PBS for 5 min six times. A secondary antibody (biotinylated goat anti-rabbit IgG) is added, and the sections are incubated at 37 °C for 40 min, washed with PBS for 5 min six times, incubated with a universal tertiary antibody (ready-to-use) at 37 °C for 40 min, and washed with PBS for 5 min six times. DAB staining is performed, followed by hematoxylin counterstaining of the nuclei, acidification, return to blue, dehydration, clearing, and mounting.

### 4.7. Real-Time Fluorescent Quantitative PCR

Total RNA was extracted from each group using the chloroform extraction method, and the concentration was quantified by nucleic acid quantification. The extracted RNA was then reverse-transcribed into cDNA. Subsequently, the PCR amplification reaction was performed. The reaction system (20 μL) consisted of TB Green Premix Ex Taq II 10 μL, forward and reverse primers (10 μM) each 0.8 μL, 50× ROX Reference Dye 0.4 μL, DNA template 2 μL, and RNase-free H_2_O 6 μL. The reaction program included a 30 s pre-denaturation at 95 °C, followed by 40 cycles of 5 s denaturation at 95 °C, 34 s annealing at 60 °C, 1 min extension at 60°C, and 15 s denaturation at 95 °C. The specific product of the quantitative PCR was analyzed by melting curve analysis. GAPDH was used as an internal control, and the relative expression of the target gene mRNA was calculated using the 2^−ΔΔCT^ method.

### 4.8. Western Blotting

Tissue samples from the cortices of mice in each group were collected and added to RIPA lysis buffer with protease inhibitors, then thoroughly ground in a low-temperature freezer mill. After centrifugation at 4 °C to obtain the supernatant, protein concentration was quantified using the BCA method, and equal amounts of protein were loaded onto the gels. Samples were separated by SDS-PAGE gel electrophoresis and transferred onto PVDF membranes. The membranes were washed with TBST and then blocked with a rapid blocking solution for 5 min. Subsequently, the membranes were incubated overnight at 4 °C with primary antibodies against HMGCR (1:5000), ACAT1 (1:5000), CYP46A1 (1:5000), NRIP1 (1:500), NR3C1 (1:1000), PSD-95 (1:1000), OCCLUDIN (1:1000), CLAUDIN-5 (1:1000), SYN (1:10,000), GAPDH (1:20,000), and TUBULIN (1:40,000). After washing the membranes with TBST three times, they were incubated with secondary antibodies at room temperature for 1 h. The membranes were again washed with TBST three times, and the bands were visualized using a LI-COR Odyssey scanner. The relative expression levels of the target proteins were calculated using Image J 1.8 software.

### 4.9. Statistical Methods

The NHANES data were analyzed using SPSS 21.0 software. Categorical variable data were expressed as rates (%) and analyzed using chi-square tests. Numerical variable data were expressed as mean ± standard deviation (x ± s) and analyzed using the Shapiro–Wilk test for normally distributed data and non-parametric tests for non-normal data. Some indicators were converted into categorical variables based on reference ranges. The Forward: LR method was used to include variables, and the Hosmer–Lemeshow goodness-of-fit test was applied to assess the model’s fit. Independent risk factors were identified, and the results of the logistic regression model were presented as OR and 95% CI, with statistical significance set at *p* < 0.05. All other data were analyzed and plotted using GraphPad Prism 9 (GraphPad Software, Inc., La Jolla, CA, USA), with mean ± SEM used to represent the data, and comparisons between groups were made using *t*-tests or one-way ANOVA, with statistical significance set at *p* < 0.05.

## 5. Conclusions

In summary, this study, set in the context of depression, took brain cholesterol metabolism as its starting point. Using NHANES data, we prospectively analyzed the relationship between human cholesterol-related indicators and depression. By employing bioinformatics, pathology, and molecular biology techniques, we identified differentially expressed small molecular markers in stress-induced injury. From a new perspective, we explored the molecular interaction between stress, cholesterol metabolism, and depression. We clarified the mechanism of action of the NR3C1/NRIP1/NR1H2 signaling pathway in the depressive-like behavior induced by short-term stress. However, the causal relationship between serum cholesterol levels and brain cholesterol efflux remains unclear. The mechanism of action of NR3C1/NRIP1/NR1H2 in the depressive-like behavior induced by long-term stress also requires further research.

## Figures and Tables

**Table 1:** Single factor analysis of depression occurrence.

Non-Adjusted Model	OR	95% CI	*p*-Value
Age			0.334
Gender	1.749	1.592~1.921	0.000
Race			0.101
Educational Level	0.867	0.836~0.899	0.000
Body mass index (BMI)	1.027	1.021~1.032	0.000
Family income	0.773	0.749~0.798	0.000
High-Density Lipoprotein (HDL)	0.994	0.991~0.997	0.000
Total Cholesterol	1.002	1.001~1.003	0.001

Note: odds ratios (OR) and confidence intervals (CI).

## Data Availability

The datasets generated during and/or analyzed during the current study are available from the corresponding author on reasonable request.
